# Treatment of Vulvovaginal Candidiasis—An Overview of Guidelines and the Latest Treatment Methods

**DOI:** 10.3390/jcm12165376

**Published:** 2023-08-18

**Authors:** Małgorzata Satora, Arkadiusz Grunwald, Bartłomiej Zaremba, Karolina Frankowska, Klaudia Żak, Rafał Tarkowski, Krzysztof Kułak

**Affiliations:** 1 Student’s Scientific Association at the I Chair and Department of Gynaecological Oncology and Gynaecology, Medical University of Lublin, Staszica 16 Str., 20-081 Lublin, Poland; arek.grunwald.2@wp.pl (A.G.); zaremba.bartek28@gmail.com (B.Z.); k.frankowska10@gmail.com (K.F.); zakklaudia3@gmail.com (K.Ż.); 2 I Chair and Department of Gynaecological Oncology and Gynaecology, Medical University of Lublin, Staszica 16 Str., 20-081 Lublin, Poland; rafaltar@yahoo.com (R.T.); krzysztof.kulak@gmail.com (K.K.)

**Keywords:** vulvovaginal candidiasis, vagina, azoles, oteseconazole, ibrexafungerp, immunotherapy, recurrent vulvovaginal candidiasis

## Abstract

Vulvovaginal candidiasis (VVC) is a common condition associated with discomfort in affected women. Due to the presence of different forms of the disease, diverse treatment regimens are developed; the newest ones include oteseconazole and ibrexafungerp. Here, we focus on the most up-to-date recommendations regarding VVC treatment, as well as novel treatment options. Topical and oral azoles are the drugs of choice in uncomplicated mycosis. The efficacy of probiotics and substances such as TOL-463 and chlorhexidine is indicated as satisfactory; however, there are no relevant guidelines. Although the majority of researchers agree that the treatment of non-albicans VVC should be long-lasting, the recommendations are inconsistent. Another clinical problem is the treatment of VVC with azole intolerance or resistance, for which literature proposes the use of several drugs including oteseconazole, ibrexafungerp, and voriconazole. The treatment schedules for recurrent VVC include mainly fluconazole; however, alternative options such as immunotherapeutic vaccine (NDV-3A) or designed antimicrobial peptides (dAMPs) were also described. We also focused on VVC affecting pregnant women, which is a substantial challenge in clinical practice, also due to the heterogeneous relevant guidelines. Thus far, few precise recommendations are available in the literature. Future studies should focus on atypical VVC forms to elucidate the inconsistent findings.

## 1. Introduction

Vulvovaginal candidiasis (VVC) is a significant public health challenge—in the United States, it affects 70–75% of women over the course of their lives, resulting in 1.4 million outpatient visits per year, while the cost of treating the disease annually reaches USD 368 million [1]. VVC remains one of the most common reasons for appointments with gynecologists and obstetricians [2]. Fungal infections are one of the leading causes of inflammation of the vagina and vulva, right after bacterial infections [1,3].

Many factors can promote or even induce VVC, such as local defense mechanism dysfunctions, gene polymorphisms, allergies, antibiotics, serum glucose levels, psychosocial stress, estrogens, and sexual activity [4]. The predominant species colonizing the vagina are *Candida albicans* and non-albicans species, such as *C. glabrata*, *C. tropicalis*, *C. krusei*, and *C. parapsilosis* [5]. The most common etiological agent causing fungal inflammation is *Candida albicans*, but infections caused by other strains are also possible. *Candida* species as a part of the vaginal flora for unknown reasons change from a commensal organism to a pathogenic one causing vulvovaginal candidiasis [6]. The potential mechanisms favoring *Candida* vaginal colonization and the host factors enhancing the diversion to infection often raise the question of whether clinicians should administer treatment or not. VVC is divided into uncomplicated and complicated cases. Uncomplicated ones are sporadic episodes of mild infections caused by *C. albicans*. Complicated cases are severe infections caused by non-*albicans Candida* species, recurrent VVC, VVC during pregnancy, or VVC associated with other medical conditions such as immunosuppression or diabetes [7]. 

Fluconazole remains the first line treatment for VVC, improving quality of life in 96% of women; however, even 63% of women have ongoing infections after completing maintenance therapy [8,9]. Discomfort and anxiety of women with permanent vaginal yeast colonization inadequately treated and serious clinical concerns regarding subsequent therapeutic decisions have been observed [10,11].

Considering the high prevalence of VVC, vaginal yeast colonization after standard treatment of VVC, and adverse consequences of topical azole antifungal agents, VVC remains a public health concern [12]. Recently, two new drugs characterized by higher efficacy in the treatment of VVC and less potential to cause side effects have appeared—oteseconazole and ibrexafungerp [13,14]. This necessitates conducting a literature review in order to summarize and evaluate treatment guidelines regarding vulvovaginal candidiasis and the need for therapeutic intervention. The aim of this paper is to present guidelines and new treatment options for vulvovaginal candidiasis.

## 2. Materials and Methods

The studies cited in this narrative review were selected from the PubMed, Google Scholar, and Scopus databases from January 2013 to June 2023. The keywords used for the search included: “vulvovaginal candidiasis”, “symptoms and diagnosis”, “uncomplicated vulvovaginal candidiasis”, “complicated vulvovaginal candidiasis”, “recurrent vulvovaginal candidiasis”, “azole intolerance”, and “vulvovaginal candidiasis during pregnancy”. We included original papers and case reports. Articles not written in English, conference abstracts only, and duplicated papers were excluded.

## 3. Symptoms and Diagnosis of VVC

In order to plan the appropriate treatment, it is crucial to collect the medical history based on the symptoms reported by the patient. The most common symptoms reported by patients are vulvar itching, pain, and sometimes also dysuria or dyspareunia, and abnormal vaginal discharge, which may be cheese-like, watery, or minimal [15,16,17,18,19,20,21,22,23]. In a study by Yano et al., the most common symptoms were itching (91.2% of respondents), burning (68.3% of respondents), and redness (58.1%) [15]. In the same study, patients also described vaginal discharge as quite thick, white, and curd-like (55.6%) [15]. 

In physical examination, vaginal discharge, erythema of the vulva and vagina, and, less frequently, abrasions or cracks of the vulva or vagina are noticeable [15,17]. Despite the frequent symptoms associated with VVC reported by women, making a diagnosis of VVC based solely on clinical presentation is not reliable. A study by Aniebue et al., conducted on 209 patients, found that only the clinical diagnosis of VVC is associated with a false positive rate. VVC occurred in 17.7% of patients based on symptoms and laboratory findings. Comparing the microbiological diagnosis to the clinical diagnosis, its sensitivity was 70.3% and the specificity was 83.7% [24]. Therefore, clinicians should be aware of the high rate of false-negative results of clinical diagnosis, which should prompt the use of microbiological and laboratory tests to confirm the diagnosis of VVC in a patient. 

Recommendations and guidelines require the collection of a vaginal swab for testing, pH assessment, odor test, and wet-mount microscopy [24,25]. A normal vaginal pH (below 4.5) together with the clinical symptoms may suggest VVC in a patient. In turn, a fishy smell or the presence of an amine will be characteristic of bacterial vaginosis or trichomoniasis [19,20]. Farr et al., in their 2021 recommendations, described that a microscopic examination of the vagina with saline or 10% KOH using light or phase-contrast microscopy can be necessary to confirm *Candida* infection. The effect of 10% KOH is to disrupt the cellular material covering the mycelium and yeast, and thus to make them visible [26]. However, it should be borne in mind that in the case of too few microorganisms, the sensitivity of microscopy will be very low [26,27]. A diagnosis of VVC can be made in the presence of noticeable budding yeast or hyphae/pseudohyphae with a polymorphonuclear to epithelial cell ratio of less than 1 [28,29].

Although microscopy has been the standard used for years, culture still seems to be the gold standard [24,30]. However, cultivation of *Candida* species takes approximately 48–72 h, which can lead to a delay in the correct diagnosis and treatment of a patient. For this reason, many clinicians make a diagnosis only on the basis of microscopy or symptoms reported by the patient, which may also result in unnecessary treatment [31].

The method of molecular testing, which is gaining in popularity, seems to be worth mentioning. Gaydos et al. conducted a cross-sectional study that showed significant a benefit from using PCR to diagnose BV, trichomoniasis, and VVC. A total of 1740 patients were qualified for the study. For swabs obtained by the reference method (isolation of potential *Candida* microorganisms from culture media), vaginal candidiasis was diagnosed in 32.8% of patients. For VVC, the sensitivity was 90.9% and the specificity was 94.1% for *Candida*, and for *C. glabrata*, the sensitivity and specificity were 75.9% and 99.7%, respectively [32].

Molecular testing may indeed hold the key to correctly identifying VVC, but future research should focus on strains such as *C. glabrata*, which are less common *Candida* species but still a problem due to treatment resistance. Therefore, the identification of a diagnostic method helpful in the diagnosis of treatment-resistant *Candida* should be the main goal of further studies on molecular testing.

## 4. Treatment of Uncomplicated VVC

### 4.1. Conventional Treatment Methods for Uncomplicated VVC

Uncomplicated VVC accounts for up to 90% of all cases of candidiasis [33]. The mainstay of treatment for most cases of uncomplicated VVC is azole antifungals, which, by inhibiting the fungal enzyme CYP51, prevent the accumulation of fungitoxic sterols [11,34]. A review of the literature from 2020 does not indicate a difference in the effectiveness of clinical treatment of VVC between oral and vaginal azole drugs. However, it should be taken into account that oral administration of drugs is usually associated with more severe possible systemic side effects than topical application; therefore, future studies may show differences in the use of these two methods [35]. Treatment with topical azoles usually lasts 3 days, and symptoms should resolve within 2–3 days after. Various topical antifungal agents are available, such as clotrimazole, miconazole, and butoconazole, but they may cause side effects such as itching and burning [1,7]. Therefore, according to the 2021 Centers for Disease Control and Prevention guidelines, uncomplicated VVC should be treated with topical preparations of short duration. This method seems to be more beneficial for patients than oral azoles because topical agents will not cause systemic side effects. Oral azoles can cause abdominal pain, headaches, and nausea. In addition, patients may be at risk of drug interactions with other medications. However, it is important to remember that topical agents may cause burning or irritation, but compared to the side effects of oral azoles, these effects do not appear to be significant [36]. Oral treatment with fluconazole consists in administering a single dose of 150 g of the drug. However, fluconazole may cause side effects such as hepatotoxicity, cytochrome P450 interactions, and possible fetal harm in pregnant women. Moreover, in approximately 50% of patients treated with fluconazole, VVC recurs after 6 months [37]. Thus, the American Society of Infectious Diseases recommends that patients should use topical and oral azoles concurrently [38].

### 4.2. Unconventional Treatment Methods for Uncomplicated VVC

Probiotics seem to be a controversial alternative to azoles. In vitro studies have shown that exogenous Lactobacilli inhibit the formation of *C. albicans* biofilm [39]. In 2021, Stabile et al. conducted a study on 40 women divided into two groups. The first group consisted of patients treated with topical clotrimazole for 6 days, and the second group of patients was treated with oral clotrimazole which contained live strains of *Saccharomyces cerevisiae*, melatonin, and *Lactobacillus idophilus* GLA-14 (Unilen^®^ Microbio+). *C. albicans* infection was found in 85% of women, and *C. glabrata* was the cause in the remaining women. Treatment with Unilen^®^ Microbio+ has been shown to be more effective than topical treatment with clotrimazole alone (90% vs. 80%). No patient experienced side effects during treatment. Moreover, the frequency of relapses was twice as high in the group treated with clotrimazole alone than in the group treated with the preparation [40]. However, the guidelines of the Centers for Disease Control and Prevention clearly indicate the lack of evidence for the use of probiotics in the treatment of VVC [36].

TOL-463 is a vaginal anti-infective based on boric acid and fortified with ethylenediaminetetraacetic acid (EDTA). In 2019, Marrazzo et al. evaluated the safety and efficacy of TOL-463 in the form of a gel or insert in the treatment of VVC. Finally, 106 women were qualified for the study, including 53 with bacterial vaginosis (BV), 36 with VVC, and 17 with both BV and VVC. As for patients with VVC, *C. albicans* was identified in almost all patients. One hundred four patients received one dose of TOL-463 in the form of a vaginal gel or insert. For BV patients, 81% of patients treated with the gel and 59% of patients treated with the insert were cured. The percentage of VVC patients cured with the insert was 92%, and the percentage of VVC patients cured with the gel was 81%. The mean time to resolution of symptoms was 7.0 days. Nineteen percent of the study participants had side effects, mostly vaginal and vulvar burning. Both methods were found to be effective and without any serious side effects in patients. Moreover, it seems to be significant that in the case of BV, 58% of gel-treated patients and 41% of insert-treated patients required additional treatment. For patients with VVC, only 13% of gel-treated patients and 8% of insert-treated patients required additional treatment [41]. Therefore, it seems that treatment with TOL-463 will be more effective in the treatment of uncomplicated VVC than in the case of BV. 

Chlorhexidine is an orally taken topical antiseptic. In 2021, Mirzaeei et al. showed the effectiveness of chlorhexidine in patients with VVC and BV. The results of the study evaluated the use of chlorhexidine in the treatment of VVC and BV compared to clotrimazole and metronidazole. A total of 111 patients were enrolled in the study—34 with VVC, 41 with BV, and 36 with non-specific vaginitis. Patients with VVC alternatively used clotrimazole vaginal cream or chlorhexidine gel. Patients with BV used chlorhexidine gel or metronidazole tablets. On the other hand, patients with non-specific vaginitis used chlorhexidine gel or clotrimazole vaginal cream with metronidazole tablets. The most common side effect was vaginal burning. In the group of patients using chlorhexidine gel, vaginal burning occurred in 14 patients with VVC, 10 patients with BV, and 23 patients with non-specific vaginitis. None of the patients reported nausea or vomiting. In the group of patients using clotrimazole gel, vaginal burning occurred in 14 patients with VVC and 16 patients with BV. Regarding oral metronidazole, vaginal burning was reported by two patients with BV. Most patients improved, and their level of satisfaction was higher than that in the group of patients using clotrimazole. Improvement was reported by 15 VVC patients using chlorhexidine gel, 16 VVC patients using clotrimazole gel, 23 BV patients using chlorhexidine gel, and 16 patients with non-specific vaginitis also using chlorhexidine gel. In this study, the effectiveness of chlorhexidine in vaginal infections was therefore higher than that of clotrimazole [42]. The studies describing new potential medical treatments for uncomplicated VVC are presented in Table 1.

Despite the effectiveness of the treatment of uncomplicated VVC with azoles, due to the potential systemic adverse effects associated with the use of oral drugs, it seems important to strive for the implementation of new methods of treatment for vaginal fungal infections. On the one hand, the studies we described showed that probiotics, TOL-463, and chlorhexidine were associated with significant effectiveness or a small number of side effects. On the other hand, the guidelines of the Centers for Disease Control and Prevention do not recommend the use of these drugs in the treatment of VVC [36]. Therefore, it is necessary to conduct further studies, which may result in the approval of these drugs for use.

## 5. Treatment of Complicated VVC

### 5.1. Treatment of VVC Caused by Non-Albicans Species

Treatment of non-albicans species VVC is challenging because they are very often resistant to azoles and nearly 50% of affected women might present minimal or no symptoms [36].

Currently, in the case of the *C. glabrata* infection, according to German guidelines from 2021, the local administration of nystatin or ciclopiroxolamine may be considered [26]. In the event of recurrence, according to the guidelines of the American Centers for Disease Control and Prevention, it is worth considering the use of therapy including 600 mg of boric acid in a gelatin capsule administered vaginally once daily for 2 weeks [1]. Nevertheless, it should be taken into account that the application of boric acid can impair fertility and might be embryotoxic; therefore, it is not recommended for women of reproductive age [26]. What is more, in the study conducted by Philipps between 1995 and 2004, 10 patients identified with non-albicans (9 with *C. glabrata* and 1 with *C. tropicalis*) infection were treated with 50 mg amphotericin B vaginal suppositories for 14 days, and after that, 8 of them showed no further infection (2 who failed were infected with *C. glabrata*) [43].

With regard to other species of *Candida*, *C. dubliniensis* is sensitive to imidazoles [44]. As for *C. tropicalis* and *C. guilliermondii*, they can be successfully treated as *C. albicans* infections, and *C. kefyr* is unlikely to cause vaginitis [44].

Along with research on the new possibilities of treating VVC appeared new hopes for reducing the treatment time for complicated episodes, thereby decreasing the risk of *Candida* strains developing resistance to the drugs. Oral ibrexafungerp exhibits in vitro activity against a broad range of *Candida* species, including echinocandin- and azole-resistant isolates [45]. However, it was reported that the drug has lower potency against *C. krusei* than against other species [46].

### 5.2. Treatment of Vulvovaginal Candidiasis in Patients with Diabetes Mellitus

Another example of complicated VVC is associated with diabetes mellitus. Both patients struggling with the disease and those with an increased risk of developing it are in the group at higher risk of VVC [26]. Possible reasons are connected with hyperglycemia in vaginal cells. It is associated with increased fungal adhesion and binding and also reduces the ability of neutrophils to perform effective phagocytosis of these cells [15,47]. 

What is more, these patients are more likely to have non-albicans *Candida species* infections, particularly those caused by *C. glabrata* and characterized by a less effective response to antimycotic therapy [26]. This may be due to the fact that some diabetic patients are treated with SGLT2 inhibitors (e.g., dapagliflozin and canagliflozin), which are supposed to increase the number of VVC episodes [26]. This is probably related to the mechanism of action of these drugs, which is based on lowering blood glucose levels by decreasing the renal threshold for glucose and increasing its excretion in the urine, which may predispose to more frequent infections.

### 5.3. Treatment of VVC in HIV-Positive Patients

As for HIV infection, there is no evidence that HIV-positive women respond worse to therapies used in HIV-negative patients, and there is no proof that VVC predisposes to HIV infection [48]. It is known that cases of VVC are less frequent than oropharyngeal candidiasis; however, if they occur in women with advanced immunosuppression, VVC episodes may be more severe and recur more often [49]. In most cases, VVC in these patients is uncomplicated and should be treated according to general guidelines as uncomplicated VVC. In the available literature, there are also no data indicating that there would be a need to delay treatment with antiretroviral therapy (ART) until the treatment for candidiasis is completed [49].

### 5.4. Treatment of VVC with Azole Intolerance or Resistance

Long-term use of oral fluconazole and/or topical clotrimazole may increase the likelihood of the emergence of azole-resistant *Candida* species. The incidence of VVC caused by treatment-resistant species such as *C. tropicalis*, *C. glabrata*, *C. krusei*, *C. parapsilosis*, *C. kefyr*, and *C. lusitaniae* is estimated at 25–45% [50]. The type of resistance and virulence of *Candida* in response to conventional VVC treatment is mainly dependent on the formation of the fungal biofilm. A biofilm is a group of microorganisms attached to a surface and surrounded by an extracellular matrix, which reduces the susceptibility of pathogens to antimicrobial agents [51,52]. Therefore, due to differences in their pathogenicity and profiles of resistance to current antifungal drugs, studies describing new therapeutic strategies for azole-resistant *Candida* can be observed in recent times.

**Figure 1:**
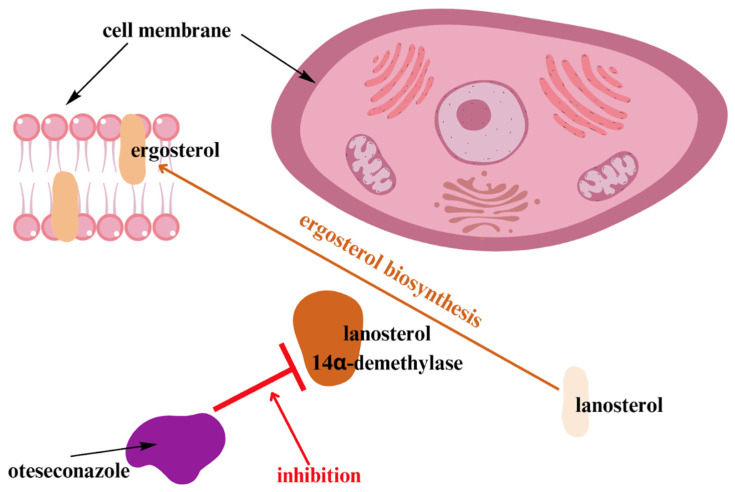
Oteseconazole—mechanism of action [53]. Oteseconazole, similar to triazoles, inhibits the fungal lanosterol 14α-demethylase and, as a result, inhibits ergosterol biosynthesis. Nevertheless, in contrast to triazoles, oteseconazole has not three, but four nitrogen atoms in a five-member ring. This results in greater selectivity for fungal enzymes [53].

Oteseconazole, approved by FDA, is a new oral azole drug that has the same CYP51 selectivity as other drugs in this class, but it does not bind to and inhibit human CYP51 without causing many side effects. The mechanism of action of oteseconazole is shown in Figure 1.

A study has shown that oteseconazole is effective not only against *C. albicans*, but also against *C. glabrata* [54]. In a 2022 multicenter, randomized, double-blind phase III study, participants were randomized to receive either oteseconazole or fluconazole. The study showed that oral oteseconazole 600 mg on day 1 (4 × 150 mg) and 450 mg on day 2 (3 × 150 mg) was as effective as fluconazole in the treatment of an acute episode of VVC, followed by oteseconazole 150 mg once daily weekly for the first 11 weeks of the maintenance phase. A phase IIa study by Brand et al., conducted on 55 participants, comparing oteseconazole to fluconazole showed no statistical difference between the groups treated with these drugs. The doses of oteseconazole were 300 mg daily for 3 days, 600 mg daily for 3 days, and 600 mg twice daily for 3 days, and the dose of fluconazole was 150 mg once daily for 3 days. The minimal inhibitory concentration (MIC) of oteseconazole against fluconazole-resistant *C. glabrata* was 64-fold lower than the MIC of fluconazole [55]. Another sensitivity study, which was conducted on 219 patients, showed that the effects of oteseconazole on fungal isolates ranged from ≤0.0005 to >0.25 μg/mL compared to fluconazole, which ranged from <0.06 to 32 μg/m^2^ [56]. The limitation of both studies is the small number of participants from whom *C. glabrata* was isolated—11.8% and 1.8% of patients, respectively [55,56]. The studies describing the use of oteseconazole in the treatment of VVC are presented in Table 2.

Ibrexafungerp, a triterpenoid antifungal medicine, reduces the number of (1,3)-β-D-glucan polymers, which weakens the fungal cell wall and leads to the death of the fungal cell. When treated with ibrexafungerp, there is not as much potential for cytochrome P450 interactions (increased with azoles) as the target only exists in the fungal cell wall [45]. In addition, most side effects are mild and mainly affect the gastrointestinal tract [57]. In a 2022 study, Schwebke et al. evaluated the effectiveness of treating patients with acute VVC with ibrexafungerp compared to a placebo group. Out of 188 patients included in the research sample, only 11 women in mycological examination showed the presence of *Candida glabrata*. The study actually showed an improvement in clinical cure rates compared to the placebo group (50.5% vs. 28.6%). However, it is limited by too few patients with strains other than *C. albicans* [57]. Therefore, more research should be conducted on the efficacy of ibrexafungerp in VVC in patients with azole-resistant strains.

Voriconazole is a second-generation triazole drug available in intravenous and oral formulations. In 2022, Morris et al. reported the efficacy of oral voriconazole with or without concomitant medications in 11 patients with refractory VVC. The voriconazole regimen consisted of 400 mg twice daily for 1 day followed by 200 mg twice daily for 13 days. Fluconazole-resistant VVC was confirmed by microscopic examination. Of the 11 isolates, 10 were fluconazole-resistant, 1 exhibited intermediate fluconazole resistance, and 10 exhibited complete or intermediate resistance to voriconazole. After 2 weeks of treatment with voriconazole, eight patients were cured. During the treatment, six women did not experience any side effects. Two women reported visual disturbances, resolving after the first days of treatment. One woman experienced nausea, muscle pain, and thinning hair. One woman experienced tingling around the mouth after 5 days of treatment, which resolved within 24 h after taking the last dose of voriconazole. The study showed that voriconazole, alone or in combination with topical agents, may be effective in the treatment of refractory VVC [58].

The increase in resistance of many *Candida* species to current antifungal drugs and the use of unproven VVC treatment methods by patients are important reasons and premises for further studies on the effectiveness of oteseconazole, ibrexafungerp, and voriconazole, as well as other new therapeutic strategies. This is important not only because of the high incidence of VVC in women, but also to improve patient comfort.

### 5.5. Treatment of Recurrent VVC

RVVC is a chronic, difficult-to-treat, devastating infection affecting women worldwide. In the United States, RVVC is defined as three or more episodes of symptomatic VVC in less than one year [59]. On the other hand, European guidelines and guidelines from the Infectious Diseases Society of America define RVVC as four or more symptomatic episodes of VVC during a one-year period [47]. Current treatment protocols confirm that at least three symptomatic episodes of VVC are considered sufficient for the diagnosis of RVVC [1]. The global prevalence of the disease is 3871 per 100,000 women, and 372 million women are affected for their entire lifetime [10]. Less common species such as *C. glabrata*, *C. dubliniensis*, *C. lusitaniae*, *C*. *tropicalis*, *C. krusei*, *C. kefyr*, and *C. parapsilosis* may be involved in the etiology of RVVC [6,60]. Vaginitis caused by strains other than *C. albicans* is more resistant to treatment [6,60]. 

The most common treatment schedule for RVVC is induction therapy with a topical antifungal drug or oral fluconazole at a dose of 150 mg for 10–14 days, and then oral fluconazole is given at a dose of 150 mg for 6 months. Resistance to fluconazole is observed in women with RVVC, but improper use of the drug must be excluded before resistance can be diagnosed [47,61]. Long-term treatment with fluconazole is associated with high costs and side effects, while about 50% of women experience a recurrence of symptoms a few months after the end of treatment [61]. Therapeutic approaches also suggested include changing the method of contraception from hormonal to mechanical ones, treating the sexual partner, and using topical gentian violet [6]. Topical treatment of RVVC may include clotrimazole, miconazole, terconazole, and vaginal boric acid, as well as nystatin. However, the literature suggests that azoles are more effective than nystatin [62]. The limited efficacy of treatment with these methods indicates the importance of implementing new, more effective therapies for RVVC that can be used in the long term without significant side effects.

Oteseconazole (VT-1161) was approved in April 2022 by the US Food and Drug Administration (FDA) for the treatment of RVVC in women without reproductive potential [56]. For women with RVVC, oteseconazole may probably be a first-line drug due to its prevention of vaginal recolonization for a longer period of time than current treatment options, especially in women infected with *C. glabrata*, as well as those with badly controlled diabetes mellitus in whom there is an impaired response to fluconazole [63]. 

**Figure 2:**
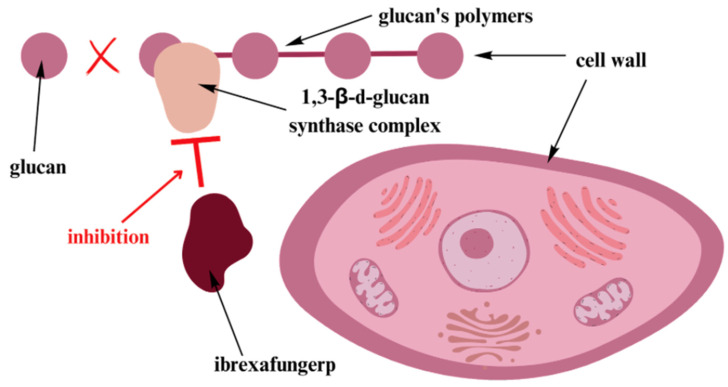
Ibrexafungerp—mechanism of action [53]. Ibrexafungerp inhibits the production of 1,3-β-d-glucan through non-competitive inhibition of the 1,3-β-d-glucan synthase complex. It results in cell instability and eventual lysis. The mechanism of action is similar to that of echinocandins; nevertheless, they are structurally different [53].

In June 2021, ibrexafungerp, a new oral glucan synthase inhibitor, was approved by the FDA for the treatment of RVVC in women after menopause [64]. It is active against multiple strains of *Candida* that are resistant to azoles and echinocandins. Figure 2 illustrates the drug’s mechanism of action. 

The recommended dose for oral administration is 300 mg (150 mg twice a day) [64]. It shows fungicidal activity in a concentration-dependent manner against *Candida* spp., and the in vitro activity does not change with azole resistance [64]. Phase III clinical trials have shown that ibrexafungerp is effective in inducing a complete clinical response with sustained resolution of symptoms compared to placebo in patients with acute VVC [57]. The studies describing the use of ibrexafungerp in the treatment of VVC are presented in Table 3.

An immunotherapeutic vaccine (NDV-3A) containing a recombinant *C. albicans* adhesin/invasin protein was evaluated for the prevention of RVVC in an exploratory, phase 2, randomized, double-blind, placebo-controlled trial. The study was conducted on 188 women with RVVC aged 18–55 years, using an approved method of birth control, who presented a clinically diagnosed, active episode of VVC at the time of study eligibility [65]. A single intramuscular dose of the vaccine was safe; it generated a rapid and robust immune response and reduced the incidence of symptomatic RVVC episodes for up to 12 months in 188 women with RVVC [47]. Despite the promising results, the efficacy of the vaccine was evaluated only according to symptoms, and a prolonged efficacy evaluation was missing. The study also did not include an assessment of patients’ quality of life, so some potential clinical measures of efficacy were not obtained [65]. Further studies are required to confirm the safety and efficacy of immunotherapeutic vaccines in RVVC.

Designed antimicrobial peptides (dAMPs) are artificially synthesized molecules modeled on naturally occurring antimicrobial peptides that have direct fungicidal activity. A 2019 study by Woodburn et al. evaluated the efficacy in vitro of four types of dAMPs (RP504, RP544, RP556, and RP557) derived from tachyplesin I for their potential use in the topical treatment of RVVC. These peptides showed broad-spectrum antifungal activity against 46 clinical isolates, including fluconazole-sensitive and -resistant strains of *C. albicans*, *C. glabrata*, *C. tropicalis*, and *C. parapsilosis*, as well as spontaneously resistant *C. krusei* [60]. Showing fungicidal activity, in contrast to fluconazole’s fungistatic effect, and having a low predisposition to induce resistance among microorganisms, dAMPs may become potential therapeutic agents for the treatment of RVVC. 

Due to the limitations of conventional treatments and personal preferences, patients with RVVC are increasingly turning to complementary and alternative medicine (CAM)—up to 40% of women with RVVC use CAM to treat or prevent VVC despite the wide availability of antifungal agents [6]. CAM includes such products as tea tree oil, garlic, probiotics (*Lactobacillus*), and agents that acidify the vaginal environment [60]. Despite the increased popularity of herbal topical preparations, such treatment methods for VVC are not recommended [1]. According to experts, given the frequent co-occurrence of *Candida spp*. with Lactobacilli in the human vagina, the use of Lactobacilli as probiotics may not be logical [47]. However, a randomized trial from 2019 by Russo et al. showed the effectiveness of a Lactobacilli mixture in combination with lactoferrin in reducing symptoms and preventing RVVC recurrence [66]. To date, good-quality evidence on the efficacy of complementary and alternative treatments for RVVC is limited, so they are not recommended [1,6].

### 5.6. Treatment of VVC in Pregnant Women

The occurrence of VVC is strongly dependent on the current hormonal state of the patients [67]. All conditions related to elevated estrogen levels, such as pregnancy and the use of hormonal contraceptives or hormonal replacement therapy, are VVC risk factors [10,68]. It is estimated that during pregnancy the incidence of VVC significantly increases in comparison to that in non-pregnant women; however, the exact statistics are missing [67]. The treatment of VVC in pregnancy is quite problematic, as on the one hand, it is definitely needed to avoid complications arising from the infection, and on the other hand, therapy schemes must be appropriately modified to make treatment the safest [69].

Currently available guidelines are largely consistent when it comes to the usage of azoles in the therapy of VVC in pregnant women [26,36]. In general, the use of locally administered azoles is recommended [26,36,48]. The guidelines developed by the Centers for Disease Control and Prevention included a limited statement recommending the use of topical azoles for 7 days, and no other detailed recommendations were established [36]. Contrastingly, the British Association for Sexual Health and HIV listed several treatment schemes which can be applied, and the distinct management for acute and recurrent VVC among pregnant women was distinguished. In case of acute VVC, the authors recommend the use of 500 mg of clotrimazole in pessary daily for 7 days. Moreover, as an alternative, they indicate a 7-day therapy regimen with the use of clotrimazole at a reduced dose (200 mg) or 5 mg of 10% clotrimazole in cream. Additionally, other treatment options include the use of 150 mg of econazole in pessary, as well as miconazole in cream (5 g of 2% agent, daily for 7 days) or in capsules (1200 or 400 mg daily for 7 days). In turn, in the case of the presence of RVVC, the guidelines recommend only the following management: topical imidazole for 10–14 days and 500 mg clotrimazole in pessary once a week for maintenance [48].

According to the latest guidelines prepared by the previously mentioned German research group, the most desirable treatment scheme is in line with the previously mentioned schemes and includes the use of clotrimazole locally. The authors especially highlight the reasonableness of such management in the first pregnancy trimester to avoid oral azole administration and the following potential fetal malformations [26]. Although the results of meta-analysis regarding the occurrence of complications resulting from fluconazole therapy indicated that such a treatment regimen did not significantly increase the risk of various congenital malformations, they suggested the need for further investigation of a potential association between heart defects and fluconazole exposure [70]. 

Several national and international guidelines recommend the application of boric acid as a substitute for azole-based therapy in non-pregnant women; however, in pregnant individuals, such a treatment regimen may be associated with an increased risk of various congenital malformations, and thus it seems that it should be avoided [71].

Another treatment option that was discussed in terms of the treatment of pregnant women affected by VVC was the use of dequalinium chloride (DQC), known for its antimicrobial and antifungal properties. Mendling and colleagues have indicated this agent as an effective treatment option leading to the cure of VVC in pregnant patients; however, their conclusions were drawn based on the results of an unpublished drug utilization trial, as well as a study with a small sample size. That is why their findings can not be considered as certainly relevant [72].

Apart from the evaluation of the role of conventional treatment methods for VVC, there are also studies concerning the usage of alternative ones based on substances of natural origin.

Abdelmonem et al. decided to compare the efficacy of the vaginal-administered mixture consisting of bee honey and yogurt with vaginal-administered tioconazole at a dose of 100 mg in the treatment of VVC in 129 pregnant women. Interestingly, among the patients in the first group, the authors noticed a greater clinical improvement in terms of itching, discharge, and vulvovaginal redness. Additionally, the application of this natural mixture resulted in a significantly lower incidence of reported treatment-related symptoms in the form of local irritation. Thus, the results of this study indicate the possibility of the application of natural-derived substances locally in VVC treatment [73]. Studies describing non-azole-based treatment methods in VVC affecting pregnant women are presented in Table 4.

Another substance that has clinical utility in the treatment of VVC accompanying pregnancy, namely redcore lotion, was assessed in a recently published meta-analysis. This name describes an extract derived from hawthorn seeds characterized by a high content of phenols, aldehydes, and ketones which is widely used in Chinese medicine due to its inhibitory action on bacteria and fungi. In evaluated studies, the effectiveness of miconazole alone as well as in combination with redcore lotion was assessed, and pregnant women represented slightly more than 60% of all participants. In the cohort of pregnant patients, the use of combined therapy showed significant improvement in reducing the symptoms of the infection and the presence of fungal cultures, both as separate and accumulative variables, when compared to the application of miconazole alone. Additionally, in the group treated with combined therapy, lower levels of adverse effects were observed [74].

Thus, considering satisfactory effectiveness, as well as a minor risk of side effects of such natural-product-based therapies, further experiments should also focus on these kinds of agents.

## 6. Discussion

The analysis of the literature on the treatment of VVC has led to many questions. Which of the latest methods of treatment seems to be the best for the management of VVC? Can any of the newly developed methods replace those already available?

VVC is still a significant problem that affects the quality and comfort of everyday functioning of patients. Although oral fluconazole is the treatment of first choice in patients with uncomplicated VVC, it still seems to be ineffective against VVC caused by species other than *C. albicans* [50]. As both the oral and topical use of fluconazole may be associated with side effects in patients, new drugs are being studied, including oteseconazole and ibrexafungerp. Studies show that these drugs are effective against treatment-resistant *Candida* species and provide a shorter treatment time than the use of fluconazole. In 2021, Sobel et al. showed that oteseconazole achieves control of VVC symptoms up to 50% faster than fluconazole [63]. In addition, the study authors showed that oteseconazole could prevent vaginal recolonization for up to 12 months. Confirmation of this hypothesis by further studies may lead to a crucial change in the treatment of patients with RVVC and comorbidities such as diabetes or HIV, which are associated with an increased probability of VVC morbidity. As for ibrexafungerp, preclinical studies have shown that in the treatment of VVC caused by treatment-resistant species, it has the same or even higher in vitro activity compared to echinocandins [75]. It seems that both oteseconazole and ibrexafungerp may be a breakthrough in the treatment of RVVC. However, it seems important to conduct clinical trials to determine the dosage of these drugs.

Despite the fact that many drugs for VVV are available on the market, there are still no officially approved vaccines or immunological therapies that can be used in antifungal treatment. The resistance to antifungal drugs acquired by patients along with the duration of treatment and the long duration of their use are a premise for conducting research on the potential use of vaccines against *Candida* infections. To our knowledge, the 2018 study by Edwards et al. is the first study to evaluate the effectiveness of the NDV-3A vaccine in the treatment of RVVC. The use of the vaccine in patients was not associated with any serious side effects. It seems important that patients with RVVC strongly responded to NDV-3A vaccination, which indicates the generation of an immune response against *Candida*. However, it also seems relevant to the results of this study that the patients over 40 years of age did not respond as well to the NDV-3A vaccine as the other patients, and the reason for this seems to be unclear. While, to our knowledge, there are currently no studies on the impact of menopause and reduced estrogen levels on the effectiveness of vaccination, the available research is on the progressive failure of the immune system with age. Perhaps the reasons for the worse results in patients over 40 who took NDV-3A should be sought in the progressive aging and approaching menopause, which is a factor for weakened immune reactions in women [76]. Therefore, further research is needed not only on immunotherapy with NDV-3A in perimenopausal patients, but also on women using menopausal hormone therapy (MHT). It seems important to determine the effective treatment in this group of patients with VVC. The age and condition of patients who would benefit from potential NDV-3A vaccination should also be determined. 

Although the available studies did not show any adverse effects resulting from the use of probiotics, their use in the treatment of VVC still seems to be illogical. Perhaps probiotics may actually be effective in women with VVC as a supplement to basic therapy. The results of a systematic review of the use of probiotics in VVC patients showed that the addition of probiotics can enhance the effectiveness and effect of conventional antifungal drugs in terms of short-term improvement in cure rates. However, probiotics used alone have no effect on long-term improvement in cure rates [77]. Moreover, the results of the Shenoy and Gottlieb study from 2019 also did not show that probiotics could have a beneficial effect on VVC or RVVC [78]. In conclusion, it seems meaningless and illogical to use probiotics in patients with VVC. Future studies should focus on determining the optimal duration of therapy with probiotics in patients with VVC and in immunocompromised patients. Moreover, studies should also focus on immunocompromised women as a result of comorbidities that were excluded from the study group in previous studies. This is important as it will determine efficacy and safety in this population.

Due to the increased risk of developing VVC during pregnancy [68] and the side effects of azoles, the treatment of pregnant patients with VVC can still be challenging. Treatment of VVC in pregnant women is necessary because it can prevent a lower birth weight of the fetus or a premature delivery [69]. While studies have not shown an increased risk of birth defects as a result of the use of 150 mg of fluconazole during pregnancy, a possible teratogenic effect is possible with 400 to 800 mg of fluconazole daily [26]. For this reason, vaginal miconazole or clotrimazole may be a safer treatment. Moreover, perhaps future research should focus on natural medicinal products, such as a mixture of bee honey and yogurt with thioconazole or redcore balm. The use of these products resulted in a significant reduction in symptoms in pregnant patients with VVC [73,74].

However, the studies reviewed in this article had several limitations. Firstly, it cannot be overlooked that most of the studies concerned phase II or III clinical trials, which makes it difficult to assess possible efficacy, other indications, or the exact dosing regimen. Secondly, studies often did not take into account patients who were immunocompromised due to comorbidities, which can lead to a poorer assessment of the effectiveness of a given drug. It seems important to conduct research on drugs such as oteseconazole or ibrexafungerp in the context of a larger group of patients with various etiologies of VVC. Moreover, the collection of patient databases from many large centers will make it easier in the future to establish a specific dosing regimen for these new drugs.

## 7. Conclusions

A review of the current literature concluded that there is a great need for the treatment of VVC infections, especially those caused by yeasts other than *C. albicans*. Currently, the greatest challenge for researchers and clinicians is the introduction of an effective and efficient treatment for RVVC. Although oteseconazole or ibrexafungerp seem to be effective in relapses, more clinical trials or treatment regimens for these drugs still need to be conducted. Implementation of vaccination against VVC may prove to be extremely important, but before that, it is necessary to determine which group of women will benefit most from such a method. It seems illogical to use probiotics because of their ability to only improve cure rates in the short term. Treatment of VVC should be individualized, as this condition may contribute to the deterioration of patients’ daily functioning.

## Figures and Tables

**Table 1:** Studies describing new potential medical treatments for uncomplicated VVC.

Author of the Study	Year of Publication	Medical Treatment	Dose	Number of Patients	Time until Symptoms Subside	Side Effects
Stabile G et al. [40]	2021	Unilen^®^ Microbio+	1 tablet containing *Saccharomyces cerevisiae* in the morning and 1 tablet containing melatonin and GLA-14 in the evening.	40	15 and 30 days	Microscopic wet-mount analysis at 1 and 3 months showed an increase in *Lactobacillus* count and a reduction in the polymorphonucleate cells in the Unilen^®^ Microbio+ group.
Marrazzo JM et al. [41]	2019	TOL-463	Medication was administered vaginally once nightly for 7 days as either a 5 g dose of gel or a 2 g unit-dose insert.	36 patients with VVC	9–12 days	Headache, vulvovaginal burning sensation, vulvovaginal pruritus.
Mirzaee S et al. [42]	2021	Chlorhexidine	Clotrimazole vaginal cream or 0.5% chlorhexidine vaginal gel was administered.	34 patients with VVC	5 days	Vaginal burning, nausea, vomiting, cutaneous lesions.

**Table 2:** Studies describing the use of oteseconazole in the treatment of VVC.

Author of the Study	Year of Publication	Number of Patients	Dose	Time until Symptoms Subside	Side Effects
Brand SR et al. [55]	2021	55	300 mg once daily of VT-1161 for 3 days, 600 mg q.d. for 3 days, or 600 mg twice daily for 3 days or receiving a single dose of fluconazole 150 mg.	28 days	Infections: nasopharyngitis, urinary tract infection, vaginitis bacterial, nausea.
Martens MG et al. [56]	2022	219	600 mg of oral oteseconazole on day 1 (4 × 150 mg) and 450 mg on day 2 (3 × 150 mg), with matching placebo capsules, or 3 sequential oral doses of fluconazole.	2 weeks	Urinary tract infection, bacterial vaginosis, headache, nausea, diarrhea, upper respiratory tract infection, pyrexia.

**Table 3:** Studies describing the use of ibrexafungerp in the treatment of VVC.

Author of the Study	Year of Publication	Number of Patients	Dose	Time until Symptoms Subside	Side Effects
Schwebke et al. [57]	2022	366	Patients were randomly assigned 2:1 to receive ibrexafungerp (300 mg twice per day) or placebo.	25 days	Treatment-related diarrhea, nausea, vomiting, dizziness, pneumonia, bronchial hyperactivity.
Grant LM et al. [64]	2022	1	Ibrexafungerp 375 mg twice daily for 3 days, followed by 375 mg twice daily on day 14.	7 days, but patient’s symptoms recurred prior to day 14 of this regimen	Fatigue, nausea.

**Table 4:** Studies describing non-azole-based treatment methods in VVC affecting pregnant women.

Author of the Study	Year of Publication	Number of Patients	Dose	Time until Symptoms Subside	Side Effects
Mendling et al. [72]	2015	60	10 mg of dequalinium chloride.	No data	No side effects.
Abdelmonem et al. [73]	2012	129	Mixture of bee honey and yogurt—30 g twice daily for 7 days.	25 days	Soiling of underclothes; local irritation.

## Data Availability

Not applicable.
